# Biopharmaceutical Study of Triamcinolone Acetonide Semisolid Formulations for Sublingual and Buccal Administration †

**DOI:** 10.3390/pharmaceutics13071080

**Published:** 2021-07-15

**Authors:** Marta Márquez Valls, Alejandra Martínez Labrador, Lyda Halbaut Bellowa, Doménica Bravo Torres, Paulo C. Granda, Montserrat Miñarro Carmona, David Limón, Ana C. Calpena Campmany

**Affiliations:** 1 Department of Pharmacy and Pharmaceutical Technology and Physical Chemistry, Faculty of Pharmacy and Food Science, University of Barcelona, Av. Joan XXIII 29-31, 08028 Barcelona, Spain; mmarquezv@ub.edu (M.M.V.); alexandraml1080@hotmail.com (A.M.L.); halbaut@ub.edu (L.H.B.); domebravo12@hotmail.com (D.B.T.); paulogranda92@gmail.com (P.C.G.); minarromontse@ub.edu (M.M.C.); 2 Department of Pharmacology, Toxicology and Therapeutic Chemistry, Faculty of Pharmacy and Food Science, University of Barcelona, Av. Joan XXIII 29-31, 08028 Barcelona, Spain; davidlimon@ub.edu

**Keywords:** triamcinolone acetonide, buccal administration, semisolid formulations, thixotropic behaviour, lidocaine hydrochloride, Franz-type diffusion cells

## Abstract

The mouth can be affected by important inflammatory processes resulting from localized or systemic diseases such as diabetes, AIDS and leukemia, among others, and are manifested in various types of buccal sores typically presenting pain. This work focuses on the design, formulation, and characterization of four semisolid formulations for oral mucosa in order to symptomatically treat these painful processes. The formulations have two active pharmaceutical ingredients, triamcinolone acetonide (TA) and lidocaine hydrochloride (LIDO). The formula also contains, as an excipient, Orabase^®^, which is a protective, hydrophobic, and anhydrous adhesive vehicle, used to retain or facilitate the application of active pharmaceutical ingredients to the oral mucosa. After designing the formulations, an analytical method for TA was validated using HPLC so as to achieve reliable analytical results. Franz-type diffusion cells were used to perform drug release studies using synthetic membrane, and permeation studies using buccal mucosa, estimating the amount and rate of TA permeated across the tissue. Additionally, sublingual permeation studies were carried out to evaluate a scenario of a continuous contact of the tongue with the applied formulation. Permeation fluxes and the amount of TA retained within sublingual mucosa were similar to those in buccal mucosa, also implying anti-inflammatory activity in the part of the tongue that is in direct contact with the formulation. In addition, the dynamic conditions of the mouth were recreated in terms of the presence of phosphate buffered saline, constant movement of the tongue, pH, and temperature, using dissolution equipment. The amount of TA released into the phosphate buffered saline in dynamic conditions (subject to being ingested) is well below the normal oral doses of TA, for which the formulation can be considered safe. The formulations applied to buccal or sublingual mucosas under dynamic conditions permit the successful retention of TA within either tissue, where it exerts anti-inflammatory activity. The four formulations studied show a pseudoplastic and thixotropic behavior, ideal for topical application. These results evidence the potential of these topical formulations in the treatment of inflammatory processes in the buccal mucosa.

## 1. Introduction

The mouth can be affected by important inflammatory processes resulting from localized or systemic diseases such as diabetes, AIDS and leukemia, among others, which can manifest in various types of buccal sores, such as canker sores or lichen planus, conditions that typically present inflammation and pain [1]. Additionally, although the oral cavity has its own bacterial flora, a qualitative and quantitative imbalance of this ecosystem leads to infections that also cause inflammatory reactions.

The treatment of oral lesions including inflammation may include lipophilic glucocorticosteroids with immunosuppressive and anti-inflammatory activity [2], such as triamcinolone acetonide (TA), at concentrations ranging from 0.05% to 0.5%, or fluocinolone acetonide at concentrations 0.025–0.05%, either in aqueous solutions or in Orabase^®^. Antiseptic agents such as chlorhexidine gluconate can be included as a mouthwash, and antibiotics such as doxycycline hyclate for lesions that involve infections, or topical anesthetics such as benzocaine hydrochloride for pain management [3]. For patients with recurrent lesions, such as AIDS patients, intralesional administration of TA has been used [4,5], which involves discomfort for the patient during treatment. In western European countries, semisolid formulations including TA can be found, including Aldoderma, Cemalyt, Positon, and Interderm, but none of them include local anesthetics such as benzocaine or lidocaine hydrochloride salts. A semisolid formulation including TA and lidocaine hydrochloride is marketed in the United States, but there is an overall lack of products containing both active ingredients around the world.

Buccal and sublingual administration are routes intended for the administration of drugs through the (buccal or sublingual) mucosa for achieving a local or systemic effect. Buccal and sublingual administration may involve higher bioavailability than oral ingestion, as they lack the enzymatic degradation and presystemic elimination within the gastrointestinal tract, as well as the first pass hepatic metabolism involved [6,7].

Buccal and sublingual administration shows inherent challenges according to the local environment, with saliva being the most significant influence. As opposed to the gastrointestinal environment upon oral ingestion (high volume of water and pH 1–3), the limited amount of saliva (0.5–2 mL) constantly present, as well as the controlled pH (5.5–7), limits the amount of drug that can be dissolved. In this respect, hydrophilic drugs could dissolve in saliva and be further ingested orally, whereas lipophilic drugs (such as TA) cannot be easily dissolved in the saliva, but will most likely diffuse towards the mucosa. Additionally, the composition of the sublingual and buccal tissues importantly influences the permeability of drugs. Whereas the mucosas of the gingiva and hard palate are keratinized and contain ceramides and acylceramides that act as a barrier, soft buccal and sublingual mucosas contain only small amounts of polar lipids (cholesterol sulfate and glucosyl ceramides), making them more permeable to water. Therefore, lipophilic drugs might be retained close to the site of application to perform a local anti-inflammatory activity, rather than being systemically absorbed, which could involve systemic toxicity [3].

This work is an extension of the work presented at the 1st International Electronic Conference on Pharmaceutics, 1–15 December 2020 [8], and shows the design and development of four semisolid formulations for administration in the buccal mucosa with the aim of locally treating inflammatory processes and pain in this cavity. These formulations have one or two Active Pharmaceutical Ingredients (API): triamcinolone acetonide (TA) and lidocaine hydrochloride (LIDO). TA is a synthetic lipophilic glucocorticosteroid with immunosuppressive and anti-inflammatory activity [2], whereas lidocaine hydrochloride (LIDO) is a hydrophilic local anesthetic that blocks sodium ion channels.

The formulations contain, as an excipient, Orabase^®^, which is a protective adhesive vehicle, hydrophobic and anhydrous, used to retain or facilitate the application of API in buccal mucosa. It has poor solubility and contains gelling agents that allow the adherence to the mucosa for periods of between 15 min and 2 h [9].

The aim of this research was the evaluation of the mechanical and biopharmaceutical properties of the semisolid formulations and to determine the influence of the concentration of TA or the presence and influence of LIDO on these properties. The suitability for topical application was therefore evaluated by performing rheology studies, while the amount and rate of TA that can be released from the formulation was determined. Additionally, the ability to permit the permeation of TA across either buccal or sublingual mucosa was studied using Franz cells and the amount of TA retained within the buccal mucosa, where the drug performs its anti-inflammatory activity, was calculated.

In addition, the dynamic conditions of the mouth were recreated in terms of the presence of water, constant movement, pH, and temperature, and mucosas with the formulations applied were immersed in dynamic conditions such that it was possible to assess the amount of TA that can be released to the phosphate buffered saline and thus estimate possible systemic effects after oral ingestion. The amount of TA retained within these tissues under dynamic conditions was also analyzed to assess the influence of these parameters on the amount of TA retained within the tissue.

To obtain fully reliable results from release, permeation, and retention studies, we designed and validated an analytical method using High-Performance Liquid Chromatography (HPLC).

## 2. Experiments

### 2.1. Materials

Triamcinolone acetonide (TA), Lidocaine hydrochloride (LIDO) and Liquid paraffin were purchased in Fagron (Barcelona, Spain). Orabase^®^ (Pectin 1–5%, Silica 5–10%, Sodium carboxymethylcellulose 5–10%, Paraffinum liquidum 75–100%) was purchased in Acofarma (Barcelona, Spain). Transcutol P was purchased from Gattefossé (Saint-Priest, France). Acetonitrile (MeCN) was purchased from Fisher Chemical (Barcelona, Spain). Ammonium acetate (≥98%) was purchased from Panreac (Barcelona, Spain).

### 2.2. Preparation and Composition of Formulations

Four semisolid formulations were prepared using the bioadhesive platform Orabase^®^ instead of water. For this task, TA, LIDO and liquid paraffin or mixtures were weighed and mixed in a mortar with a pestle. Then, pre-weighed Orabase^®^ (*ad* 100%) was added to the formulation in increasing amounts by mixing up. The semisolid formulations obtained were further mixed and homogenized by an Ultra-Turrax T10 basic (IKA, Staufen, Germany) and placed in glass containers (Table 1).

### 2.3. Rheological Properties

The rheological characterization of the formulas was performed in duplicate at 25 °C, using a Thermo Scientific Haake Rheostress 1 rheometer (Thermo Fischer Scientific, Kalsruhe, Germany) equipped with a cone-plate geometry (C60/2° Ti), connected to a temperature control device (Thermo Haake Phoenix II + Haake C25P; Thermo Fischer Scientific, Kalsruhe, Germany) and operated using Haake Rheowin^®^ Job Manager v. 3.3 software. The viscosity and flow curves were obtained in rotational mode by performing an ascendant shear rate ramp from 0 to 100 s^−1^ during 3 min, followed by 1 min at a constant rate of 100 s^−1^, and from 100 s^−1^ to 0 s^−1^ during 3 min.

The data obtained for each formulation were adjusted to different mathematical models: Newton, Bingham, Casson, Ostwald, Herschel-Bulkley and Cross.

### 2.4. Analytical Method Validation

The validation of the analytical method of TA using High Performance Liquid Chromatography (HPLC) was carried out in a Waters HPLC system equipped with a Waters pump 1525, a UV-vis 2487 detector (Waters, Milford, EE. UU.) and a Supercosil LC-ABZ (15 cm; 4.6 mm and 5 µm) column. The data were collected and processed using the Empower Pro software (Waters, Milford, CT, USA). The mobile phase consisted of 50:50 (*v*/*v*) water/methanol. Samples of 10 µL were injected and TA was detected at 232 nm according to a validated method for a different route of administration [10,11,12]. TA was initially dissolved in Transcutol P, and further diluted using a mixture of MeCN:Ammonium acetate buffer pH 4.7 (10:90) [13]. Six different calibration curves were made by preparing stock solutions of 205 μg/mL TA, and further dilutions of 102.5 μg/mL, 68.3 μg/mL, 41 μg/mL, 20.5 μg/mL and 10.25 μg/mL. Linearity, accuracy, precision, limit of detection (*LOD*) and limit of quantification (*LOQ*) were estimated as follows.

#### 2.4.1. Linearity and Range

Linearity of the method in the defined range of concentrations was evaluated by performing a least squares regression on the experimental data and evaluating the correlation coefficient (r) based on Equation (1):

*y* = *SB* · *x* + *a* (1)

where *x* is the concentration, *y* is the chromatographic area, *SB* is the value of the slope and *a* is the y-intercept [14].

**Figure 1:**
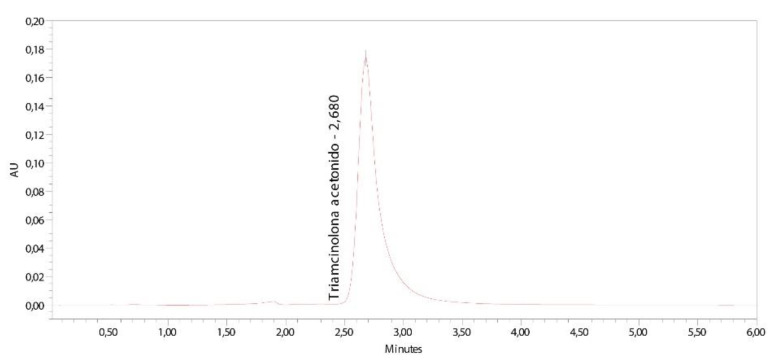
Chromatogram of the TA standard solution.

Linearity is the ability within a defined range to obtain results directly proportional to the concentrations (amount) of the analyte in the sample. The range is the interval defined by the upper and lower concentrations of the tested drug for which it has been proved that the method achieves a suitable level of accuracy, precision and linearity [15]. Figure 1 shows a typical chromatogram obtained in the analysis of samples containing TA.

#### 2.4.2. Accuracy and Precision

Accuracy at each concentration was expressed as the mean percentage deviation or relative error (*RE*, %) calculated using Equation (2):

%*RE* = [(*Cobs* − *Cnom*)/*Cnom*] · 100 (2)

where *Cobs* is the observed concentration and *Cnom* is the nominal concentration of each standard solution.

Precision was calculated and expressed as the relative standard deviation (*RSD*, %) of each replicate series, using Equation (3):

%*RSD* = (*SD*/*Cobs*) · 100 (3)

where *SD* is the standard deviation and *Cobs* is the nominal concentration.

#### 2.4.3. Determination of Limits

*LOD* and *LOQ* were calculated based on the standard deviation of the response and the slope of the calibration curve using the following equation:

*LOD or LOQ* = *K* · *SDsa*/*Sb* (4)

where *K* is a factor related to the level of confidence (3 for *LOD* and 10 for *LOQ*). *SDSa* is the standard deviation of the intercept (a) and *Sb* is the slope of the calibration line [16].

The results of the analytical method validation show that the 6 calibration lines are linear from 6.26 to 100.20 µg/mL, showing a correlation coefficient (r^2^) in the range of 0.9993–0.9998 for each line. The method is accurate and precise in the range of 6.26 µg/mL to 100.20 µg/mL, with an accuracy of 92.49% and precision of 98.23% (at 6.26 µg/mL). Finally, the *LOD* of the method was 2.63 ± 1.19 µg/mL and the *LOQ* calculated was 7.97 ± 3.60 µg/mL.

### 2.5. Release Studies

*Static conditions*: To assess the release of TA from the 4 different types of formulations, drug release experiments were performed in triplicate using Franz-type diffusion cells (FDC 400, Crown Glass, Somerville, NY, USA), with the donor and receptor chambers being separated by nylon synthetic membranes (Type NY1 μm). The receptor chambers were filled with a mixture of MeCN: Ammonium acetate buffer pH 4.7 (10:90), complying with sink conditions. The Franz-type diffusion cells were connected with a temperature-controlled circulating bath at 37 °C. The dose applied in the donor compartment was 1.5 g of formulation in a diffusion area of 2.54 cm^2^. Samples of 300 µL were collected from the receptor compartment and replaced with the same volume of fresh receptor fluid.

**Figure 2:**
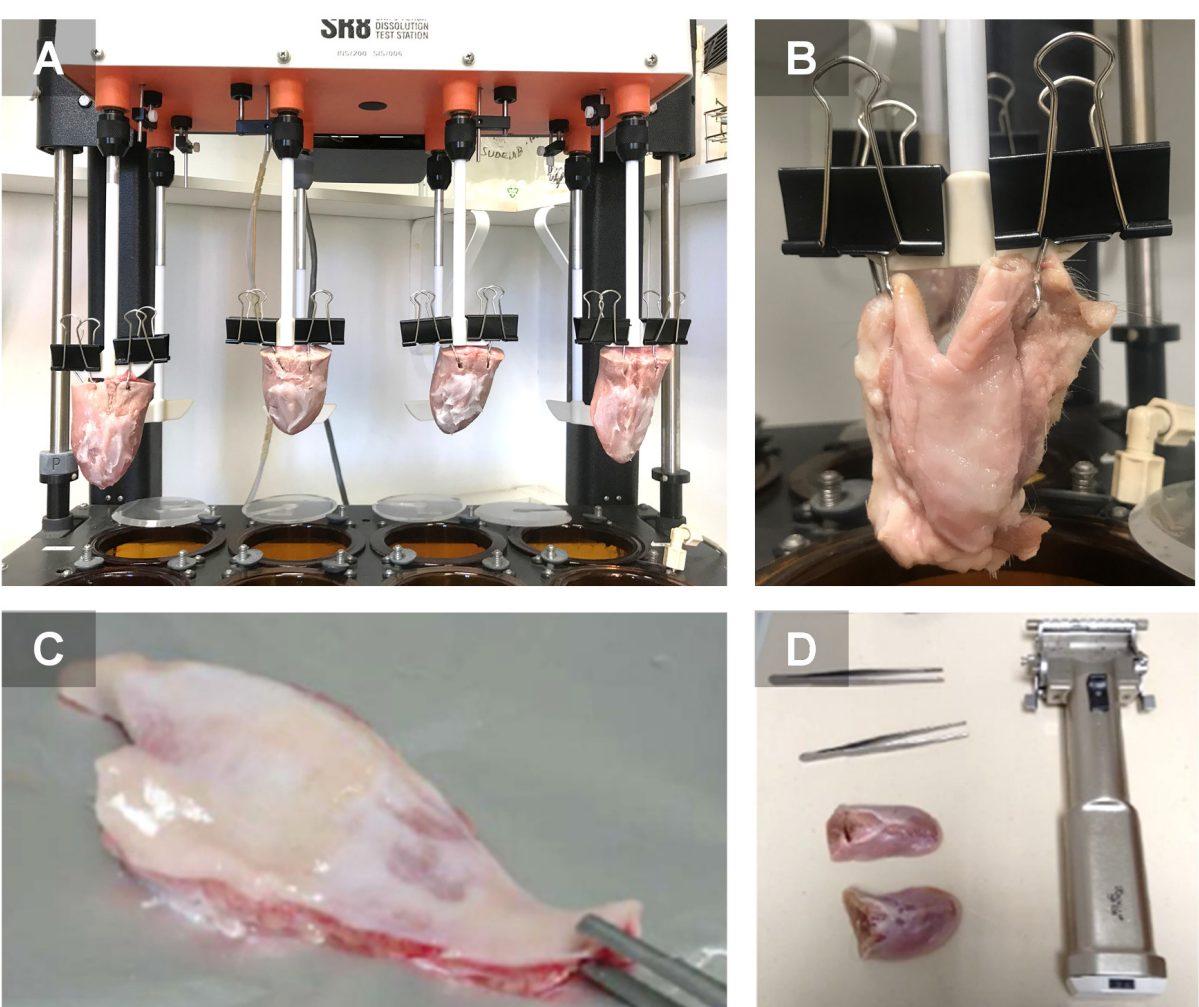
Dissolution equipment showing porcine sublingual (**A**) and buccal (**B**) mucosas for release and retention of TA studies in recreated dynamic conditions of the mouth. (**C**) Porcine buccal and (**D**) sublingual mucosas used for permeation experiments in static conditions.

*Dynamic conditions*: To assess the release of TA under recreated conditions of the mouth, a Hanson Research SR8 SRII Flask Dissolution Test Station was filled with phosphate buffered saline adjusted to a pH 6.5 as receptor fluid and the experimental conditions were adjusted to 37 °C. The sublingual (Figure 2A) and buccal (Figure 2B) mucosas (*n* = 5) with ≈2 g of the formulation applied into each were immersed in the dissolution station under constant stirring for a period of 6 to 8 h, and samples (300 μL) were collected at known intervals with the micropipette MODEL 5000 (Gilson, Middleton, WI, USA), and stored frozen in HPLC vials until analyzed. The application surface of the formulations was approximately 10 and 12 cm^2^ for the sublingual and buccal mucosas, respectively.

### 2.6. Permeation and Retention Studies

*Static conditions:* Ex vivo permeation and retention studies were conducted in Franz-type diffusion cells with a setup that is similar to that of release studies, but replacing the membrane for either sublingual (Figure 2C) or buccal (Figure 2D) porcine mucosa. The receptor fluid consisted of Transcutol^®^ P, the surface was 0.64 cm^2^. The amount of formulation applied into the donor compartment was 100 mg.

The mucosa samples were frozen at −20 °C and longitudinally cut into 700 µm slabs with a dermatome GA 630 (Figure 2B). Mucous membrane samples were placed between the receptor and donor compartments with the proximal side in contact with the receptor medium and the mucous side in contact with the donor chamber [17]. The flux values of TA (µg/h) across mucous membranes were estimated through the slope of the cumulative amount of TA permeated versus time for each formulation. Moreover, the retention of TA was estimated in the mucous membranes after the permeation experiment.

At the end of the permeation study, the amount of drug retained in the mucosa (µg/cm^2^) was extracted by ultrasound-assisted extraction. The biological samples were cleaned and washed with gauze soaked in a 0.05% solution of dodecyl sulfate and distilled water. The mucosa epithelium was removed and immersed in 4 and 2 mL of acetonitrile:transcutol (50:50 *v:v*) for the sublingual and buccal mucosa, respectively, and sonicated (100 KHz) for 30 min. The samples were filtered and quantified by HPLC.

*Dynamic conditions:* The amount of TA retained within buccal and sublingual mucosas after 8 h application in dynamic conditions was obtained with a similar procedure to that of static conditions. The receptor fluid consisted of Phosphate buffered saline adjusted to pH 6.5, the surface was 10 cm^2^ and 12 cm^2^ for sublingual and buccal mucosa, respectively. The amount of formulation applied into mucosas was 2 g.

### 2.7. Statistical Analysis

Non-parametric Student’s *t*-tests and ANOVA test were performed using a GraphPad Prism 3 (GraphPad Software Inc., San Diego, CA, USA) for comparing the different formulations.

### 2.8. Histological Observations

The integrity of the sublingual and buccal mucosas was studied under brightfield microscopy. Eight hours after application of 0.05% TA + LIDO formulation, tissues in contact with the formulation were frozen and kept until analyzed. Tissues were fixed overnight by immersion in 4% paraformaldehyde in phosphate buffer 20 mM pH 7.4, and further processed for paraffin embedding. Vertical histological sections were obtained, stained with hematoxylin and eosin and mounted under a cover slip. Samples were observed using a Leica DMD 108 optical microscope (Spain).

## 3. Results and Discussion

### 3.1. Composition of the Formulations

Four different formulations (Table 1) containing TA for topical administration were developed so as to evaluate the influence of TA concentration and the presence or absence, and influence of LIDO on the mechanical and biopharmaceutical properties of the formulation. For instance, TA was prepared at 0.05% and 0.1% (*w*/*v*), and LIDO was tested at 2% concentration. Liquid paraffin and Orabase^®^ were included as excipients for promoting the formation of a homogeneous and consistent hydrophobic film upon application in order to maximize the retention of the API in the area of application.

### 3.2. Rheological Properties

**Figure 3:**
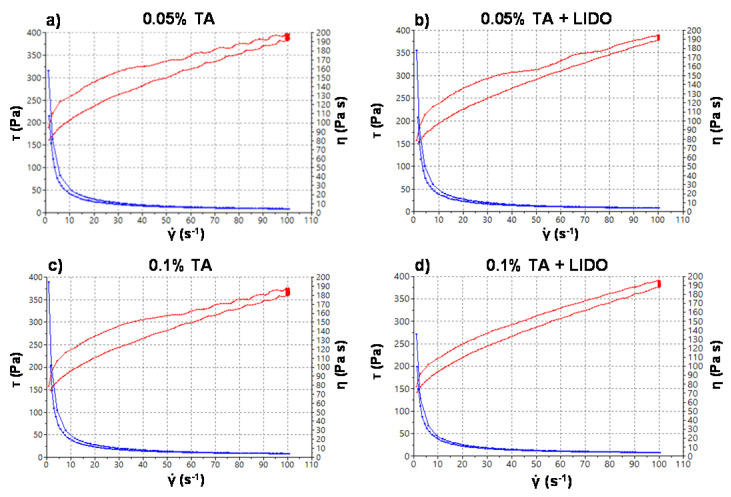
Viscosity curve (blue line) and flow curve (red line) of the 4 formulations under study. (**a**) 0.05% TA; (**b**) 0.05% TA + LIDO; (**c**) 0.1% TA; (**d**) 0.1% TA + LIDO, at 25 ± 0.1 °C.

The rheological characteristics of the formulations play an important role in physical stability, and are a crucial attribute for the development of topical drug products [18]. To find out the mechanical properties of the formulations, rheology studies were performed, and they revealed a pseudoplastic and apparent thixotropic behavior in all the formulations (Figure 3), both being desirable characteristics for topical application, allowing the formation of a consistent film covering the application area that facilitates the diffusion of the drug through the matrix [19,20,21,22].

Table 2 shows that the viscosity is similar in all formulations, except for 0.1% TA, in which it is slightly lower. On the basis of the precision of the rotational test for these kinds of highly viscous products, the lower viscosity of 0.1% TA is difficult to explain, since it was expected to be in the same range. This is presumably due to the form of disturbance of the microstructure of the gel under the effect of shear. The viscosity of our formulations was approximately eight times higher than that of a reference formula [22], because the Transcutol used as a cosolvent in the reference was not incorporated in these formulations. This fact implies that it will not confer the same adhesive force, but we can explain or predict that, with the high viscosity values found, the difference in the formula 0.1% TA will hardly translate into less adherence.

Moreover, the four formulations are shear thinning systems with the same flow behavior adjusted by Cross model (Equation (5)), for both the ascendant and descendant sections:

(5) $$\mathrm{Cross}\;\mathrm{equation}\text{: }\tau =\dot{ɣ}\cdot \frac{\eta_{\infty }+\left(\;\eta_{0}-\eta_{\infty }\right)}{1+{\left(\frac{\dot{ɣ}}{{ɣ}_{0}}\right)}^{n}\;}$$

where *τ* is the shear stress (Pa); $\dot{ɣ}$ is the shear rate (1/s); ɣ_0_ is the zero-shear rate (1/s); *η*_∞_ is the infinite shear rate viscosity; *η*_0_ is the zero-shear rate viscosity (Pa·s); *n* is a dimensionless rate constant.

### 3.3. Release Studies

**Figure 4:**
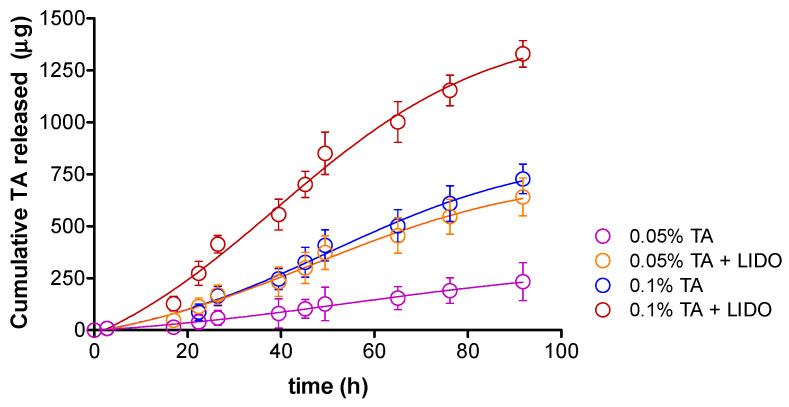
Cumulative amount (µg) of TA released versus time (h) from the four different formulations. Values represent Mean ± SD (*n* = 3).

To ensure that the TA can be released from the matrix of the pharmaceutical form and can reach the biophase, drug release studies were performed using Franz-type diffusion cells (*static conditions*). For each formulation, the cumulative released amount of TA (µg) versus time (h) was obtained in triplicate (Figure 4), all of them following a Boltzmann sigmoidal model in accordance with the coefficients of determination (r^2^) ≥ 0.98.

TA is released to a different extent depending on the formulation, after 76.2 h being released 1154.33 µg (0.08%) from TA 0.1% + LIDO, 609.11 µg (0.04%) from TA 0.1%, 546.33 µg (0.04%) from TA 0.05% + LIDO, and 190.78 µg (0.01%) from TA 0.05% formulation. Therefore, the presence of LIDO promotes a higher amount of TA released. These results might suggest that either the higher (2%) amount of Orabase^®^ in the formulations without LIDO might account for a higher retention of TA in the formulation, or that the ionic nature of LIDO, which can undergo a faster solvation and diffusion in the medium, could indirectly promote a faster release of TA. The overall low release in static conditions observed implies that it prevents the active ingredient to be released to the saliva and be swallowed. Instead, the main purpose of the formulation is to promote the penetration of TA in the tissue to act in situ.

**Figure 5:**
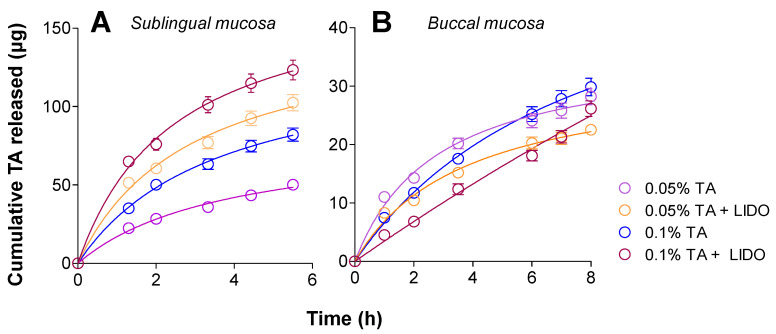
Cumulative amount of TA released (µg) to the phosphate buffered saline in dynamic conditions over time (h) when applied onto the (**A**) sublingual or (**B**) buccal mucosa. Values represent Mean ± SD (*n* = 5).

Another question is that in order to more closely estimate the amount of TA that is released from the semisolid formulation to the saliva upon application onto either buccal or sublingual mucosa, and therefore estimate the possible systemic effect due to oral ingestion of the corticoid, the conditions inside the mouth were recreated in terms of solvent, pH, temperature, and constant movement of the tongue (dynamic conditions). For this purpose, a Dissolution Test Station containing phosphate buffered saline at 37 °C pH 6.5 as receptor fluid. The mucosas (*n* = 5) with each formulation applied onto it (≈2 g) were submerged into the phosphate buffered saline and they were kept under constant stirring for 6–8 h. Samples from the receptor fluid were taken at certain intervals, and the amount of TA released (μg) was plotted as a function of time (h) (Figure 5). For both sublingual and buccal mucosas, the mathematical model that best fits the TA released is the hyperbola according to the coefficients of determination (r^2^) ≥ 0.99.

In sublingual mucosa, the amount of TA released after 6 h in dynamic conditions was 50.2 μg (0.003%) for 0.05% TA formulation, 102.5 μg (0.005%) for 0.05% TA + LIDO, 82.1 μg (0.004%) for 0.1% TA, and 123.4 μg (0.006%) for 0.1% TA + LIDO. In buccal mucosa, the amount of TA released after 6 h in dynamic conditions was 28.2 μg (0.001%) for 0.05% TA formulation, 22.5 μg (0.001%) for 0.05% TA + LIDO, 29.9 μg (0.001%) for 0.1% TA, and 26.2 μg (0.001%) for 0.1% TA + LIDO. In general, a lower amount of TA released is observed in dynamic conditions in comparison to static conditions, which could be explained by the low solubility of TA in aqueous media such as the saliva. Moreover, in both tissues a higher concentration of TA in the formulation promotes a higher release of TA. Nonetheless, the presence of LIDO has a different influence depending on the tissue. In sublingual mucosa, the presence of LIDO promotes a significantly higher TA release, similar to observations in release experiments under static conditions, whereas in buccal mucosa, the observed differences are not significant with the presence of LIDO. According to these results, the formulations that could imply a lower amount of TA released into the saliva and therefore ingested, for either sublingual or buccal application, are those with 0.05% TA. However, the amount of TA released to the receptor fluid (<125 μg) does not in either case represent a dose that could induce systemic effects upon ingestion, as oral doses of triamcinolone in adults normally range from 4 to 50 mg per day [23,24].

### 3.4. Permeation and Retention Studies

**Figure 6:**
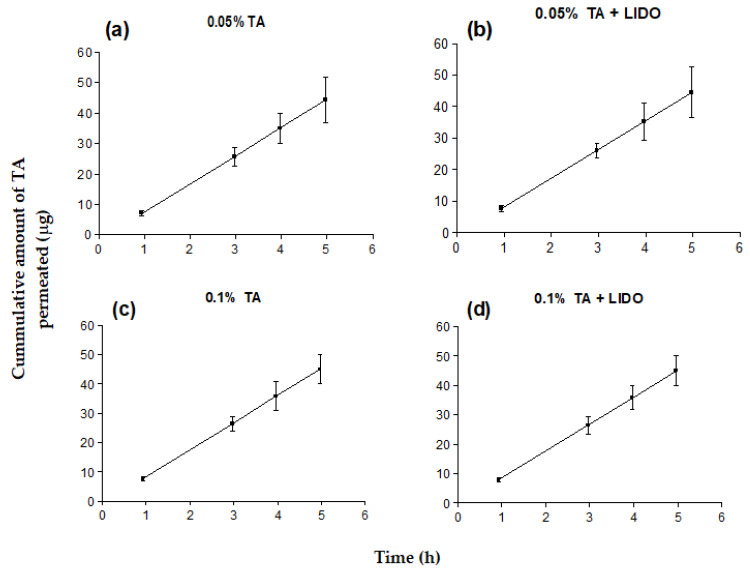
Buccal permeation kinetics of TA for the different formulations. (**a**) 0.05% TA. (**b**) 0.05% TA + LIDO. (**c**) 0.1% TA. (**d**) 0.1% TA +LIDO. Values represent Means ± SD (*n* = 5).

Ex vivo permeation studies of the four different formulations (*n* = 5) were carried out in static conditions to test the ability of TA to permeate the buccal mucosa and it being retained within the tissue upon application. The experiment setup was similar to the release studies in static conditions using Franz cells, but replacing the membrane for either the porcine sublingual or buccal mucosa. The amount of TA (µg) permeated across either mucous tissue was plotted versus time (h), and a linear least squares regression was performed (Figure 6).

The results show that TA can permeate buccal mucosa at approximately 9.2 µg/h regardless of the TA concentration or the presence or absence of LIDO (Table 3), as no significant differences were observed (>0.05) according to Student’s *t*-tests.

Considering a possible systemic effect after application of the formulations, Argenti D et al. [23] determined the multiple-dose pharmacokinetics, pharmacodynamics, and tolerability of a newly developed formulation of inhaled TA. They found that the maximum serum concentration (Cmax) at the steady state was 1.83 ng/mL. In addition, they found that TA treatment reduced the basal serum cortisol concentrations by 20% relative to the placebo treatment.

For this reason, the concentrations at a steady state (Css) for each formulation were calculated according to the permeation parameters obtained and the reported pharmacokinetic parameters of TA. For instance, upon treatment with these formulations, Css values would oscillate between 1.54 and 1.57 ng/mL, values that are 15% below those reported (1.83 ng/mL), with all formulations having similar systemic safety profiles.

**Figure 7:**
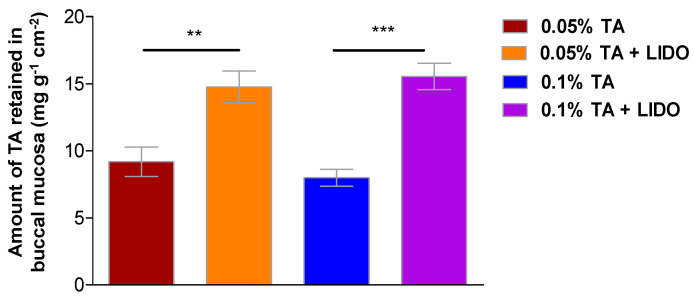
Amount of TA retained per gram and square centimeter of buccal mucosa, 6 h after application of each formulation (0.05% TA or 0.05% TA + LIDO, or 0.1% TA or 0.1% TA + LIDO). Values represent Mean ± SEM (*n* = 5). Statistical differences ** (*p* < 0.01), *** (*p* < 0.001).

The amount of TA retained within the buccal mucosa was calculated by extracting the drug from the tissue after permeation experiments with the four different formulations (Figure 7), finding that application of 0.05% TA leads to 9.2 ± 2.4 mg TA retained per gram and square centimeter of tissue, whereas application of 0.05% TA + LIDO leads to 14.8 ± 2.7 mg·g^−1^·cm^−2^, representing a 60% increase. Similarly, the application of 0.1% TA results in 8.0 ± 1.4 mg·g^−1^·cm^−2^ while 0.1% TA + LIDO results in 15.6 ± 2.2 mg·g^−1^·cm^−2^ representing a 95% increase in retained TA. Student t-tests confirmed that there is a significant increase (*p* < 0.01) in the amount of TA that can be retained in the tissue so as to perform its therapeutic activity when the formulations include LIDO, suggesting this drug also behaves as a penetration enhancer.

**Figure 8:**
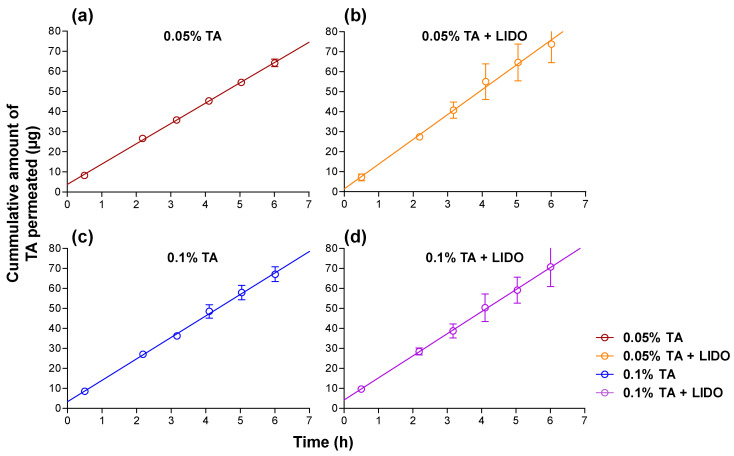
Sublingual permeation kinetics of TA for the different formulations: (**a**) 0.05% TA, (**b**) 0.05% TA + LIDO, (**c**) 0.1% TA, (**d**) 0.1% TA +LIDO. Values represent Means ± SD (*n* = 5).

In addition, permeation studies were also performed in sublingual mucosa, considering the possibility that the tongue accidentally contacts the formula, revealing whether the applied TA could still permeate in the sublingual mucosa. The cumulative permeated amount of TA in sublingual mucosa over 6 h upon application of each type of formulation (*n* = 5) was obtained (Figure 8).

Sublingual permeation also shows a linear behavior, with fluxes slightly higher than those observed in buccal permeation, ranging between 10.1 µg/h and 12.4 µg/h, as observed in Table 4. Student’s *t*-tests were performed in order to evaluate the influence of the presence of lidocaine hydrochloride in the formulations, revealing significantly higher fluxes both at 0.05% TA concentration (*p* < 0.001) and 0.1% TA concentrations (*p* < 0.05), and suggesting that LIDO behaves as a permeation enhancer in sublingual mucosa, through mechanisms of action that could include the reversible loss of integrity of the skin and mucosa barriers, the increase in the partitioning of the drug in the tissue, or the increase in the solubility of the drug [24,25]. The effects of a permeation enhancer may differ when combined with one or another drug [26].

For this reason, the concentrations at steady state (Css) for each formulation were also calculated and resulted in a range of 1.67–2.06 ng/mL, similar to the values reported (1.83 nm/mL) [23], and which could indicate some possible systemic effects in the semisolid formulations of TA.

Corticosteroids can affect keratinocytes and prevent the secretion of collagen and hyaluronic acid by fibroblasts in the dermis, interfering with cell proliferation and with long-term glucocorticoid usage. As a result, skin thinning ensues. Topical administration could produce local side effects, including skin atrophy, ecchymosis, erosions, striae, delayed wound healing, purpura, easy bruising, acne, hirsutism and hair loss [27]. Therefore, it is important to point out that these semisolid formulations should be used for short periods only, and in accordance with the medical prescription.

**Figure 9:**
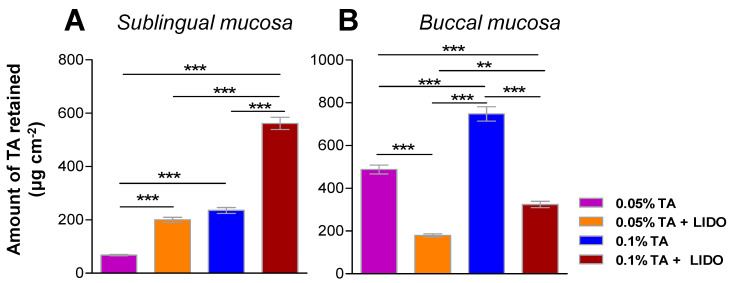
Amount of TA retained in (**A**) sublingual or (**B**) buccal mucosa 8 h after application of the formulation in dynamic conditions. Values represent Means ± SD (*n* = 5). Significant differences: ** (*p* < 0.01) *** (*p* < 0.001).

The amount of TA retained in sublingual or buccal mucosa 8 h after application of the different formulations was also studied in dynamic conditions. In sublingual mucosa, both a higher TA concentration in the formulation and the presence of LIDO promoted a higher retention of TA within the tissue (Figure 9A), a behavior also observed under static conditions. In contrast, in buccal mucosa (Figure 9B), a higher concentration of TA promotes a higher amount of TA retained within the tissue, but the presence of LIDO decreases the amount of TA retained. Overall, a higher amount of TA is retained in the mucosas under static conditions (Franz cells experiments) than under dynamic conditions (dissolution equipment), which can be explained by the higher and prolonged adherence of the formulation to the mucosa in dry, static conditions, permitting the partitioning and further diffusion towards the tissue.

### 3.5. Histological Observations

**Figure 10:**
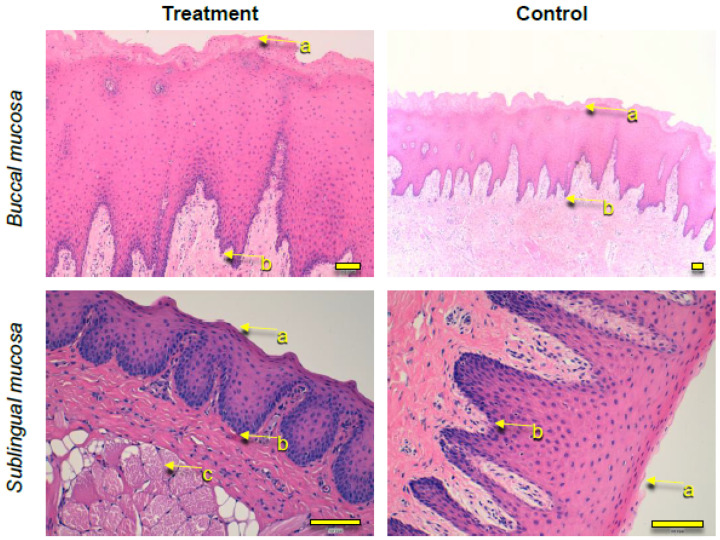
Histological observations of buccal (**top**) and sublingual (**bottom**) mucosas after application of the formulation 0.05% TA + LIDO (**left**). Untreated tissues were processed in parallel as a control (**right**). (**a**) Stratified flat keratinized epithelium, (**b**) basal layer (**c**) collagen fibers. Scalebar represents 100 μm.

Histological observations were performed with sublingual and buccal mucosa tissues after treatment with 0.05% TA + LIDO formulation. Untreated tissues were processed in parallel as a control. Vertical histological sections were obtained and treated with hematoxylin (this was to stain the nucleus) and eosin (this was to stain the cytoplasm and extracellular matrix), and were observed under brightfield microscopy (Figure 10). For both buccal and sublingual mucosas, no important differences between the untreated samples and those with the formulations were observed. The stratified flat keratinized epithelium (outermost part of the tissue) is intact after being in contact with the formulation, similar to untreated tissues. Additionally, the thickness of the dermal papilla (between the flat keratinized epithelium and the basal layer) is similar in treated and untreated tissues and no alteration of cellular structures is observed. These observations indicate that the application of the formulation does not induce any local tissue damage.

## 4. Conclusions

Formulations containing 0.05% or 0.1% TA, and in the presence or absence of 2% LIDO, were designed and developed for buccal application as potential treatments for important inflammatory processes in the buccal mucosa, such as those occurring upon buccal cancer radiotherapy, lichen planus, and canker sores, among others. The effect of TA concentration and the presence or absence and therefore the influence of LIDO on the mechanical or biopharmaceutical properties of the formulations were extensively studied. The four different formulations showed a pseudoplastic and thixotropic behavior, ideal for topical application. In static conditions using Franz cells, TA can be released from the formulations following a Boltz Sigmoidal behavior, and it was found that that the presence of LIDO promotes between 107% and 212% more TA being released after 92 h (*p* < 0.05), and that the formulation of 0.05% TA + LIDO showed the highest amount of TA released (1330 µg).

Moreover, under the recreated dynamic conditions of the mouth (constant movement, presence of phosphate buffered saline, temperature), the formulations applied onto buccal or sublingual mucosa can release small (<150 μg) amounts of TA into the phosphate-buffered saline (subject to being ingested) due to the poor solubility of TA in aqueous media, which is much below the normal oral doses of TA (4–50 mg per day), indicating no risk of systemic effects due to oral ingestion.

Additionally, permeation studies using Franz cells (static conditions) showed that TA can successfully permeate buccal mucosa at rates ranging between 9.19 and 9.24 µg/h, with no apparent influence from the concentration of TA or the presence of LIDO. After application, TA is successfully retained beneath the buccal mucosa and then performs its anti-inflammatory action, with it being found that the presence of LIDO can increase the amount of TA in the tissue by 60% or 95% (*p* < 0.01). In sublingual mucosa, TA can permeate at fluxes ranging from 10.1 to 12.4 μg/h, and the presence of LIDO enhances the permeation through the tissue.

In the recreated dynamic conditions of the mouth, TA can be successfully retained as well in the buccal mucosa (179 to 800 μg/cm^2^), observing that the presence of LIDO decreases the amount of TA retained. Provided there is a continuous contact of the tongue with the zone of application, TA could also penetrate into the sublingual mucosa, especially if the formulation contains LIDO, representing a possible anti-inflammatory and anesthetic effect both in the buccal mucosa as well as in the region of the tongue that is in contact with the formulation.

## Figures and Tables

**Table 1:** Composition of the four different formulations.

Composition	0.05% TA	0.05% TA + LIDO	0.1% TA	0.1% TA + LIDO
TA	0.05%	0.05%	0.1%	0.1%
LIDO	-	2%	-	2%
Liquid paraffin	5%	5%	5%	5%
Orabase^®^	*ad* 100%	*ad* 100%	*ad* 100%	*ad* 100%

**Table 2:** Rheological evaluation of the different formulations at 100 s^−1^. Values represent Means ± SD (*n* = 2).

Formulations	Viscosity (mPa·s) at 100 s^−1^
0.05% TA	3890.0 ± 39.8
0.05% TA + LIDO	3833.0 ± 27.9
0.1% TA	3662.0 ± 42.3
0.1% TA + LIDO	3819.0 ± 39.8

**Table 3:** Amount of TA permeated in buccal mucosa per hour (flow). Values represent Means ± SD (*n* = 5). No significant differences were observed (*p* < 0.05).

Formulations	Flow (µg/h)
0.05% TA	9.24 ± 0.03
0.05% TA + LIDO	9.19 ± 0.06
0.1% TA	9.24 ± 0.03
0.1% TA + LIDO	9.22 ± 0.02

**Table 4:** Amount of TA permeated in sublingual tissue per hour (flow). Values represent Mean ± SD (*n* = 5). Significant differences versus formulation with the same concentration of TA but without LIDO: * (*p* < 0.05), *** (*p* < 0.001).

Formulations	Flow (µg/h)
0.05% TA	10.10 ± 0.12
0.05% TA + LIDO	12.40 ± 0.42 ***
0.1% TA	10.74 ± 0.20
0.1% TA + LIDO	11.04 ± 0.14 *

## Data Availability

Not applicable.

## Abbreviations

The following abbreviations are used in this manuscript:

API	Active Pharmaceutical Ingredient
TA	Triamcinolone acetonide
LIDO	Lidocaine hydrochloride
HPLC	High-Performance Liquid Chromatography
MeCN	Acetonitrile
LOD	Limit of detection
LOQ	Limit of quantification
SD	Standard deviation
Css	Concentration at steady state
Cmax	Maximum concentration
